# Head lice in the eyelashes

**DOI:** 10.11604/pamj.2024.47.29.42559

**Published:** 2024-01-23

**Authors:** Meryem Benchekroun Belabbes, Narjisse Taouri

**Affiliations:** 1 Mohammed V Souissi University, Faculty of Medicine and Pharmacy, Rabat, Morocco

**Keywords:** Head lice, nits, eyelashes

## Image in medicine

Infection of the eyelashes with head lice is extremely rare. We report the case of a 10-year-old child who presented with a head lice infection of the eyelashes. A 10-year-old male patient presented to the ophthalmology department complaining of itching of the eyelids, redness, watering, whitish discharge, and a burning sensation for one week. The family of the patient reported that the kid had a history of recurrent episodes of head lice for which he was still treated. Visual acuity was 10/10 for both eyes. Slit lamp examination shows translucent oval structures located at the emergence of the upper eyelashes in both eyes: nits (white arrows figure A). One louse is found anchored to the eyelashes of the left eye (grey arrow figure A and B). The rest of the clinical examination including the fundus examination was strictly normal. The treatment was based on using forceps to physically remove the lice and nits from the eyelashes and the application of ophthalmic grade petrolatum ointment to the eyelids about 2-4 times in association with the treatment of the head lice. The treatment was efficient and the eyelashes were clear from both nits and lice after 10 days of treatment. Lice are hominoxius hematophagous arthropod and an obligate parasite of human beings. There are three types of lice: head lice, body lice, and crab lice. Head lice infest mainly schoolchildren and they are transmitted through close head-to-head contact. The treatment is based on the removal of the lice and nits.

**Figure 1:**
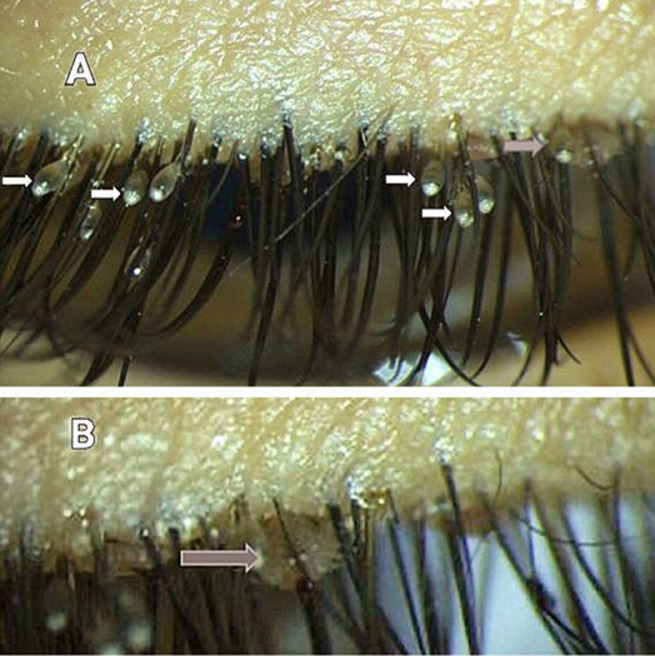
A,B) head lice, nits, eyelashes

